# The Administration of Chloroform

**Published:** 1879-09

**Authors:** Wm. Meacher

**Affiliations:** Portage, Wis.


### ARTICLE III.

### THE ADMINISTRATION OF CHLOROFORM. By WM. MEACHER, M.D., of Portage, Wis.

I believe that the administration of chloroform is not rightly understood by many medical men, and many physicians, as well as patients, are afraid of it. But it is pretty certain that most if not all of this comes from the want of a proper knowledge of its use, and there is but little doubt that nearly all the deaths which have occurred under chloroform were not due to any fault of the anæsthetic, nor to any diseased condition of the patient, such as fatty degeneration of the heart, etc., as is commonly supposed, but to faulty administration, and might have been prevented. Again, it is commonly believed that patients with disease of the lungs, or heart especially, ought not to take chloroform. Yet, as a rule, there is no objection to their taking it. I know these are broad assertions, but I am convinced that they are true. Mr. Syme used to say, that any case for operation was a case for chloroform; and Mr. Lister says, that patients with disease of the heart ought not to undergo an operation of any importance without chloroform. "It might be expected," he further says, "that chloroform in the early or exciting stage of its operation, would act upon a diseased heart like mental emotion and cause irregularity, or cessation of its contractions; this, however, does not seem to be the case, and judging from my own experience, I should say that it tends rather to remove intermission or irregularity of the pulse. On the whole I believe that chloroform, by preventing shock and mental effort during the operation, as well as anxiety before it, is in reality a great source of safety in heart disease; and if a person with known cardiac affection decides to place himself in the hands of the surgeon, so far from being unsuited for the anæsthetic, he is, before all others, the man who stands most in need of its protecting influence."

I think I am correct in saying that most physicians, in administering chloroform, direct their attention almost wholly to the pulse, while the fact is, it requires no attention at all. This is what Mr. Lister says: "The very prevalent opinion, that the pulse is the most important symptom in the administration of chloroform, is certainly a most serious mistake. As a general rule, the safety of the patient will be best promoted by disregarding the pulse altogether." It is the breathing that is to be attended to. Here lies the whole secret. Now, in watching the breathing, it is not enough to see that the respiratory movements of the chest go on—that is, expanding and contracting—but that air enters the lungs; or in other words, that the patient is really breathing; because the respiratory movements of the chest may be going on, apparently all right, yet not a bit of air going into the lungs. Right here is where the danger comes in. This is not caused, as has been supposed by the "tongue falling back," nor by paralysis of the muscles which hold up the epiglottis, allowing it to fall back as in deglutition ; but by portions or folds of the mucous membrane at the rima glottidis—properly speaking, the aryteno-epiglottidean folds—falling together, as it were, and obstructing the opening into the larynx. Mr. Lister demonstrated this by a series of experiments upon himself. This occlusion of the air passage generally occurs before the patient is fully under the influence of chloroform, and is usually preceded by stertorous breathing, but sometimes it is not, the breathing being shut off so quietly that it is unnoticed by any one except the person on the lookout for it. This the administrator should attend to, from first to last, and when it occurs, the proper thing to be done is this: Cease the administration at once, lay hold of the tongue with a napkin or a piece of cotton cloth, to prevent the fingers from slipping, or, what is better, seize the tongue with a pair of polypus forceps, or hook a tenaculum into the base of it, and draw it out steadily and quite strongly. After this traction on the tongue has been made a very short time, the air will be heard to rush into the lungs with a loud noise, similar to the inspiratory noise a person makes in snoring, when the breathing will go on again all right, and the administration may be resumed. To illustrate this point, I must quote more from Mr. Lister: "As an example of the risk that is run by want of close attention the respiration, I may mention the following case: A surgeon of considerable experience was giving chloroform to a patient on whom an operation was being performed, of which I was a mere spectator, but I noticed that stertorous breathing came on and gradually passed into complete obstruction, while the administrator was not aware of it. Seeing that the patient was in danger, I suggested to the giver of the chloroform the propriety of drawing forward the tongue ; he replied that this was uncalled for, and pointed to the heavings of the chest as evidence that the breathing was proceeding freely. Knowing that these efforts were doing nothing for the respiratory function and feeling that there was no time for discussion, I stepped out of my province so far as to seize the tongue myself and draw it forward, when a long and loudly stertorous inspiration demonstrated the necessity for the interference. Had the delusive movements of the chest been trusted to, it is probable that they might have continued until the heart had become so enfeebled by the asphyxiated state as to cause no perceptible pulse at the wrist; and had death occurred under the circumstances, the case would have been set down as one in which the heart failed first. The administrator would have been absolved from all blame, and the fatal event would have been attributed to idiosyncrasy, or to disease of the heart."

To sum up, then, a very few words will suffice for describing the proper mode of administering chloroform. Sprinkle about a drachm on the middle of a folded napkin or small towel, and hold it to the patient's nose and mouth, taking care that free space is afforded for the access of air beneath its edges; meanwhile watch the breathing closely, and if at any time it should become obstructed or strongly stertorous, suspend the administration and draw the tongue firmly forward until the obstruction to the breathing has disappeared.

At the time Mr. Lister's article, from which I have so largely quoted, was published, he said the method I have just described had been pursued at the Edinburgh and Glasgow infirmaries, two of the largest surgical hospitals in Great Britain, for nine years, without a single accident; and he concludes that, "chloroform in the hands of competent persons is a safe anæsthetic."

There is no doubt but ether is the safer anæsthetic, in the hands of those who do not rightly understand the administration of chloroform. For this reason, I suppose, and because it is an American discovery, it is the anæsthetic recommended by most of the medical colleges in this country. But ether is inferior to chloroform in several respects. It is generally much more disagreeable to the patient and troublesome to the surgeon, especially in private practice, and in operations requiring great delicacy of manipulation and perfect quiet of the parts being operated upon, such as operations upon the eye, etc. Also in obstetrical practice.

